# Agricultural land use among mestizo colonist and indigenous populations: Contrasting patterns in the Amazon

**DOI:** 10.1371/journal.pone.0199518

**Published:** 2018-07-05

**Authors:** Cristian Vasco, Richard Bilsborrow, Bolier Torres, Verena Griess

**Affiliations:** 1 Facultad de Ciencias Agrícolas, Universidad Central del Ecuador, Quito, Ecuador; 2 Carolina Population Center, University of North Carolina at Chapel Hill, Chapel Hill, United States of America; 3 Universidad Estatal Amazónica, Puyo, Ecuador; 4 Department of Forest Resources Management, the University of British Columbia, Vancouver, Canada; University of Maryland Baltimore County, UNITED STATES

## Abstract

This paper compares land use patterns of mestizo colonists and indigenous populations in the central Ecuadorian Amazon, based on data from a household survey covering mestizo colonist, Kichwa and Shuar households. As expected, colonists mostly engage in commercial agriculture and cattle ranching, but there are substantial differences in land use patterns between the Shuar and the Kichwa. The Shuar engage in cash cropping and cattle ranching, and on average, devote even more land to agricultural uses than mestizo colonists in this sample. In contrast, the Kichwa engage more in subsistence crop production and less in commercial agriculture. Such different patterns appear related to local conditions, earlier migratory and settlement patterns, and the level of exposure to markets. The implications of this for policy are explored in the conclusions.

## Introduction

As with most environmental problems, deforestation is closely linked to human activities, especially agriculture [1, 2]. In fact, agricultural expansion is widely identified as the main proximate driver of deforestation in tropical forests and is also associated with a number of other environmental problems, including desertification, soil erosion, climate change and biodiversity loss [3, 4]. Therefore, understanding the interactions between humans and the environment in the context of the socioeconomic drivers of land cover change is important for the conservation of forests as well as the other ecosystem services it provides.

Much has been written on the determinants of land use decisions in the tropics. The literature makes a clear distinction between the land use patterns of migrant colonists and those of the traditional inhabitants. Colonists are generally reported to use unsustainable production systems featured by extensive clearing of land with continuous incorporation over time of additional forest areas into agricultural production [5, 6]. This contrasts with a common belief that indigenous populations are associated with sustainable agricultural practices (long fallow, in the Boserup [7] sense) with little environmental impact and hence compatible with resource conservation [8, 9]. Nevertheless, several studies [10–15] show that indigenous peoples sometimes also engage in unsustainable practices, including cash cropping, cattle ranching and timber logging when in contact with the market economy. Such changes are a matter of concern, given the importance of large indigenous territories for conservation in Ecuador and throughout the Amazon basin [16, 17], the high rates of deforestation in colonist lands [18], the high rates of population growth in indigenous communities [12, 19], and the accelerated integration of indigenous peoples into the market economy [20].

In the case of the Amazon rainforest, one of the world’s biodiversity hotspots [21, 22], few studies have compared land use patterns of indigenous and colonist migrants. In the Peruvian Amazon, Hvalkof [23] found that the Shumahuani people keep most of their lands in forest and carry out sustainable management practices in contrast to local colonists who clear forest to establish pastures. In the Brazilian Amazon, van Vliet et al. [24] found that slash and burn cultivation system is practiced mainly by colonists while the more environmentally friendly shifting cultivation system is principally used by indigenous peoples. Nevertheless, the authors note that this difference is blurred due to increased integration of indigenous peoples into the market economy. Also in the Brazilian Amazon, Caviglia-Harris and Sills [25] found no differences in land use patterns of colonists and “traditional” farmers. The authors concluded that socioeconomic conditions matter more than cultural background when explaining land use.

Finally, several studies have been conducted on the Ecuadorian Amazon, generally concluding that indigenous practices have a much lower environmental impact than colonists [10, 26, 27]. Thus combining satellite imagery and household survey data for the northern Ecuadorian Amazon, Lu et al. [27] found that colonists managed substantially larger agricultural areas (twice the area in crops and five times the area in pasture) than indigenous populations, even though the latter had access to at least four times the area per household. The authors note, however, that there are substantial differences among indigenous communities, with some ethnic groups engaging in cattle ranching and commercial agriculture more than others. Among other studies, Rudel et al. [10] found that the Shuar–an indigenous group from the southern Ecuadorian Amazon- adopt more sustainable agricultural systems (perennial cash crops such as cacao and coffee) in contrast to colonists who engage primarily in cattle ranching. Similarly Rudel et al. [26] note that during the 1980s, colonist migrants and the Shuar responded in different ways to a pest infestation affecting a local cash crop *naranjilla*–a citrus fruit which accounted for most of the income of both ethnic groups: Colonists responded by converting infested fields to pasture, while the Shuar allowed secondary forest to develop in former *naranjilla* fields and engaged in the cultivation of customary garden crops and small areas of cash crops (coffee and cacao). But do these patterns still apply in a context of high rates of population growth and urbanization [12, 19], scarcity of land [28], increased off-farm opportunities [29], and increased integration of indigenous peoples into the market economy [20]?

This research addresses this and related questions using data from a household survey covering both colonist and indigenous (Kichwa and Shuar) households in the province of Pastaza, in the central Ecuadorian Amazon. Following this introduction, the rest of this paper is structured as follows: the next section describes the socio-economic-cultural context of the migrant colonists, the Kichwa and the Shuar in the Ecuadorian Amazon. Then the theoretical framework is presented, data collection explained and the statistical estimation methodology described. Finally, the results are presented and discussed, followed by concluding remarks and policy implications.

## The study context: Migrant colonists, Kichwa and Shuar in the Ecuadorian Amazon, focusing on Pastaza province

In the 1960s, following the enactment of Ecuador’s major land reform legislation in 1964 and the first discovery of significant oil reserves in the northern Amazon in 1967, roads were constructed by oil companies to lay pipelines to pump petroleum from the Amazon to the Pacific port of Esmeraldas for export. This led to a large migration of mostly poor farmers with little or no land, principally from the Sierra or highlands region, to the Amazon in search of land. Although in the case of Ecuador -in contrast to Brazil, for example [30], this was a process of spontaneous migration [31], it was also encouraged by the Government in an effort to occupy and incorporate the “empty lands” of the Amazon into agricultural production [26, 32]. Such migrants brought with them knowledge of production systems characterized by low technology, high dependence on family labor, cattle ranching, and exploitation of natural resources [5]. In fact, clearing a portion of forest and providing evidence of agricultural use was a precondition to claim legal rights over the plot [6], although this was never enforced. Since most soils in the Ecuadorian Amazon are not suitable for agriculture [33, 34], colonists tend to compensate for the common low fertility of Amazonian soils by clearing more land for cultivation. As a consequence, deforestation rates in the Ecuadorian Amazon rank among the highest in the world [35]. Pressures on land have intensified in recent decades as a result of high rates of population growth of colonists and the continuous process of land subdivision [30]. Furthermore, the Ecuadorian Amazon may be considered essentially a closed frontier, with little available unused land left, since large areas have been either appropriated by the State to create three large national parks or allocated to indigenous peoples via communal land titles.

**Fig 1:**
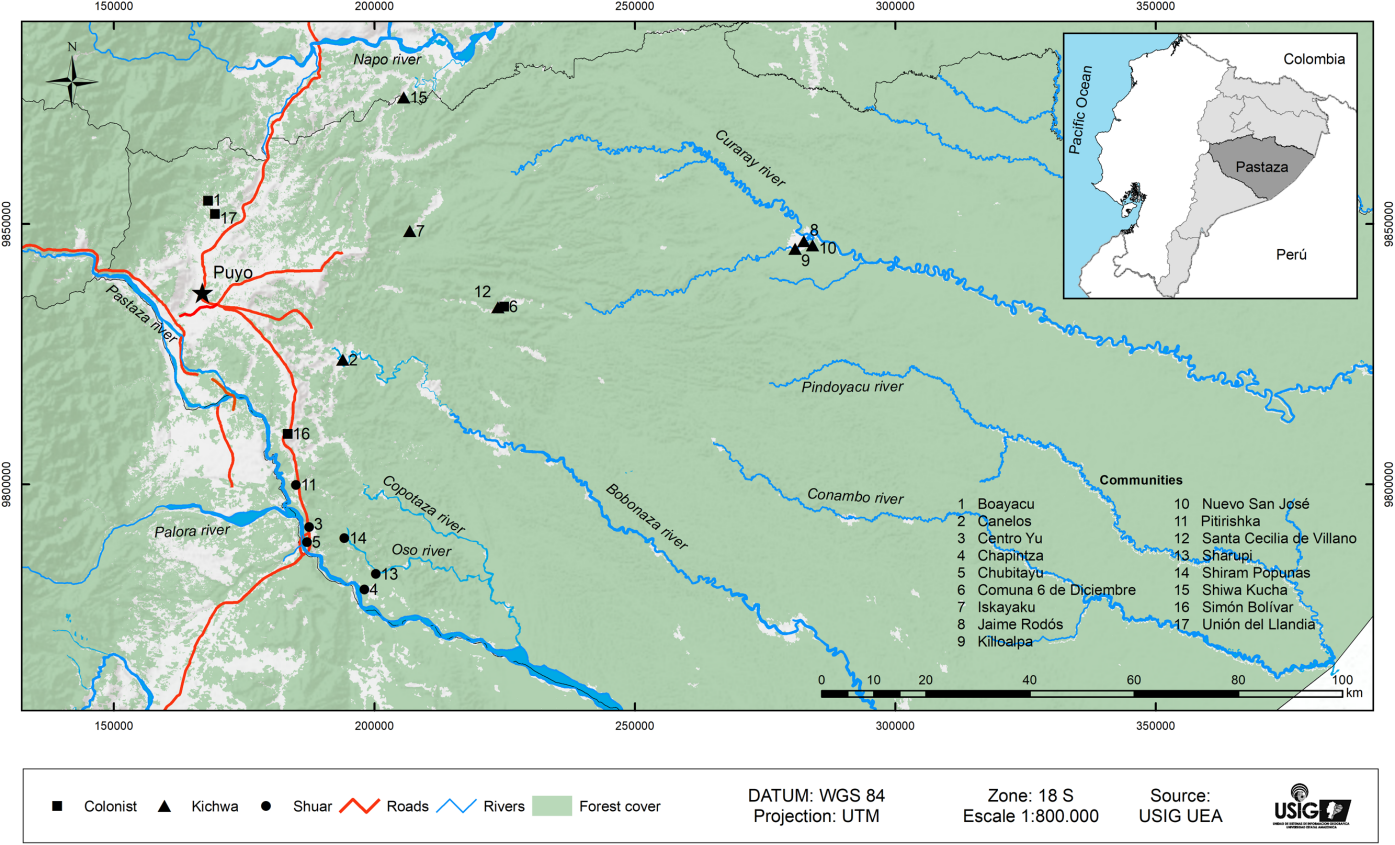
The study area and communities in Pastaza province, Ecuadorian Amazon. Reprinted from [49] under a CC BY license, with permission from Universidad Estatal Amazónica, original copyright 2017.

In terms of the main land use practices of the three study populations in Pastaza, first, mestizo colonists number about 46,000 and are settled in the westernmost part of the province, near Puyo, the provincial capital (see Fig 1). They specialize in cash crop production (*naranjilla*, cacao and sugar cane) as well as some cattle ranching [36]. In contrast, the lowland Kichwa are the most numerous indigenous group in both the Ecuadorian Amazon overall and in the province of Pastaza, with approximate populations of about 125,000 and 18,000, respectively [37]. The Kichwa of Pastaza derive from the fusion of several Amazon peoples (Shuar, Achuar, Zaparos and Andoas) as well as highland Kichwa who migrated to the Amazon to escape Spanish colonial oppression [38, 39]. Some scholars [39, 40] describe how the Kichwa obtain their livelihoods principally from subsistence farming (cassava–locally known as “*yuca*”, plantain, *taro*, and maize), the collection of forest products, hunting and fishing. Nevertheless, others report that some Kichwa have adopted colonist-style agricultural systems and nowadays also engage in commercial agriculture, cattle ranching, timber logging and off-farm wage employment in areas close to roads and urban centers [29, 41]. Although the Kichwa control extensive areas of land (approximately 1,400,000 hectares) in Pastaza under common property land entitlement regimes and with population densities generally low, pressures on land do exist in some communities where population growth is high and agricultural land is concentrated along a main river or road.

The so-called “jíbaros” or Shuar are the traditional inhabitants of the tropical forests of southeastern Ecuador, numbering about 78,000 persons, 80% in the province of Morona Santiago [37]. During the 1960s, in response to increasing colonist encroachment, they sought property rights to extensive areas of land in Morona Santiago with the support of Catholic NGOs. In order to support their claims, they replicated the colonist strategy of clearing areas of forest, planting pasture and establishing small herds of cattle [10]. They did this not only in their home territories but also in provinces to the north where some migrated to establish communities along roads. There they tended to adopt colonist’s livelihood activities, including commercial agriculture, cattle raising and off-farm employment [42]. The Shuar of Pastaza number about 5,600 and are mainly concentrated along the road going south from Puyo towards Morona Santiago (see Fig 1), their province of origin [37]. The Shuar of Pastaza hold common property rights over about 200,000 ha of land [36], with population densities higher than those in Kichwa communities [43], which may be associated with higher deforestation.

## Theoretical framework

The rural livelihood model and the microeconomic model of the agricultural household [44, 45] together provide a suitable point of departure to analyze household decisions regarding land use and the factors that affect it. The model of the agricultural household, as expanded by the livelihoods framework, hypotheses that a household makes resource allocation decisions based on the endowments of natural capital (land, water, trees), physical capital (irrigation canals, agricultural machinery and implements, roads), human capital (education, skills, health), financial capital or its substitutes (cash, credit, savings and cattle); and social capital (networks, community associations). The household is the agricultural decision-making unit which intends to maximize its total utility based upon limited endowments of capital as well as contextual factors, including physical and natural capital and alternative sources of income.

This framework has been previously used in other studies analyzing land use patterns among colonist [6, 46] and indigenous peoples [12]. Murphy et al. [6] have noted that agriculture in the Amazon tends to differ from that of most of the developing world in being based on an abundance of land compared to other factors of production (labor, capital and infrastructure). This leads farmers in the Amazon to use land-extensive and labor-saving production systems. Pichón [47] elaborated on the livelihoods model focusing on land use decisions in the Amazon rainforest, noting that household land use decisions are shaped by both household-specific and exogenous variables. Household-specific variables include endowments of land, labor and capital, in addition to demographic characteristics including household size, education endowments and other aspects of human capital. Exogenous factors encompass a) the household’s natural resource base (i.e., land area and quality/fertility of soils), and b) variables reflecting national policy and the institutional environment (availability and quality of schools and hospitals, property rights, credit, the road system, bus transportation networks, and the size of local towns and agricultural markets, among others), availability of alternative income sources (off-farm job opportunities) and access to technology (agricultural inputs and extension services). These variables determine the returns to land, labor and capital and therefore play a significant role in household decisions regarding land use [5]. With this theoretical framework, we develop a multivariate regression model to assess the effects of the household and contextual variables on agricultural land use of mestizo-colonists and the two main local indigenous peoples in the central Ecuadorian Amazon.

## Methodology

### Data and variables

The data came from a household survey conducted in May-October 2013 in Pastaza (Fig 1). A questionnaire template (available upon request) from the Poverty and Environment Network (PEN) [see 48] was adapted to obtain information on household demographic characteristics, land use, household assets, social capital, and sources of income (including off-farm employment), as well as use of natural resources. At the same time, a community survey inquiring about population size and infrastructure was completed by community leaders. The survey model was approved by the Ethics Committee at Universidad Estatal Amazónica. All interviewees were asked for oral approval before conducting the survey.

Households were selected a two-stage approach, in which the first stage was to select communities following criteria of ethnicity, distance to the nearest accessible road, distance to the nearest town of more than 10,000 inhabitants -which in all cases was Puyo- and population size and density. Variation in these conditions across communities ensures a fair representation of the diversity of communities, contributing to the robustness of the study [48].

In the second stage of the sample, households in each sample community were randomly selected from a list provided by community’s leaders, with large proportions of households selected from small communities and small proportions from large ones, yielding 8–30 households per community. The final result is that a total of 304 households (116 Kichwa, 120 Shuar and 68 colonist) were successfully interviewed in 17 communities (7 Kichwa, 6 Shuar and 4 colonist) (see Table 1). The survey was administered to the household head with assistance of spouse. Although the sample is not a strictly probability sample of communities, the 17 communities provide good representation of diverse ethnicities, geographies and diversities of livelihoods, and the households selected within them are randomly selected in Pastaza.

**Table 1:** Communities in the sample.

Community	Population	Predominant ethnic group	Accessible by	Time needed to reach Puyo (hours)
Comuna 6 de Diciembre	45	Colonist	Dirt road	3.5
Boayacu	50	Colonist	Dirt road	1.0
Unión del Llandia	180	Colonist	Dirt road	0.5
Simón Bolívar	3000	Colonist	Paved road	0.5
Centro Yu	50	Shuar	Dirt road	1.5
Shiram Popunas	141	Shuar	Trail	6.0
Sharupi	94	Shuar	Trail	3.0
Chapintsa	420	Shuar	Dirt road	2.0
Pitirishka	250	Shuar	Paved road	0.75
Chubitayu	1125	Shuar	Paved road	1.0
Iskayaku	60	Kichwa	Trail	3.0
Shiwa Kucha	310	Kichwa	Dirt road	2.0
Jaime Roldós	75	Kichwa	River	8.0
Killoalpa	75	Kichwa	River	8.0
Nuevo San José	150	Kichwa	River	8.0
Santa Cecilia	150	Kichwa	Dirt road	3.5
Canelos	1200	Kichwa	Dirt road	1.0

### Statistical methods

We use a multivariate regression approach to model land use patterns of indigenous and colonist households. The dependent variables of interest are the total cultivated area, area in staples (plantain, *yuca*, *taro* and maize), area in cash crops (mainly *naranjilla*, cacao and sugar cane), and area in pasture, all expressed as fractions of the total area available for use. These are estimated from the values reported by respondents as collected by interviewers. Previous research in the Amazon [50] shows that self-reported values are reasonably consistent with estimated obtained through using other measurement techniques (i.e., direct measurement on the ground). Additionally, the mean values of this study (see Table 2) are similar to those of prior research [27] using satellite imagery.

**Table 2:** Descriptive statistics, definitions and mean values for colonist, Kichwa and Shuar households.

Variable	Description	Overall	Colonists	Shuar	Kichwa
***Dependent variables***					
Total cultivated area	Fraction of land devoted to crops and pastures	0.35 (0.33)	0.43^b^ (0.33)	0.37^b^ (0.34)	0.25^a^ (0.31)
Staple crops	Fraction of land in plantain, cassava, taro and maize	0.11 (0.19)	0.10^a^ (0.22)	0.12^a^ (0.21)	0.11^a^ (0.17)
Cash crops	Fraction of land in *naranjilla*, cacao and sugar cane	0.01 (0.06)	0.03^a^ (0.11)	0.02^a^ (0.06)	0.01^a^ (0.04)
Pastures	Fraction of land in pasture (ha)	0.10 (0.21)	0.18^b^ (0,28)	0.16^b^ (0.23)	0.02^a^ (0.07)
***Household-level variables***					
Age	Age of household head (years)	41.6 (13.7)	45.4^ab^ (16.5)	39.7^a^ (12.6)	41.5^b^ (13.1)
Education	Completed years of formal education of head (years)	8.3 (8.45)	8.2^a^ (4.7)	9.6^b^ (4.0)	7.5^a^ (4.0)
Colonist	Household head is colonist (0/1)	0.23	-	-	-
Kichwa	Household head is Kichwa (0/1)	0.38	-	-	-
Shuar	Household head is Shuar (0/1)	0.39	-	-	-
Household size	Number of household members	4.5 (4.6)	3.7^a^ (1.8)	4.2^a^ (2.0)	5.3^b^ (2.4)
Wealth index	First principal component of household assets.	0.196 (1.834)	1.505^c^ (1.515)	0.270^b^ (1.685)	-0.838^a^ (1.763)
Off-farm employment	Household has off-farm income in previous 12 months (0/1)	0.79	0.85^b^	0.81^b^	0.64^a^
Distance from dwelling to road	Distance to closest road (km)	15.00 (32.5)	2.08^a^ (14.2)	1.74^a^ (9.78)	25.59^b^ (44.25)
Participation in social events	Times household participated in community work in previous 12 months	14.7 (15.86)	7.2^a^ (8.6)	22.1^b^ (18.1)	11.9^a^ (13.4)
Short duration of residence	Household head has resided in community ≤10 years (0/1)	0.18	0.26^b^	0.24^b^	0.07^a^
Medium duration of residence	Household head has resided in community 10–25 years (0/1)	0.25	0.36^b^	0.33^b^	0.14^a^
Long residence	Household head has resided in community > 25 years (0/1)	0.55	0.38^a^	0.42^a^	0.79^b^
Total land area available for use	Households total land (ha)	50.28 (82.13)	27.76^a^ (43.67)	45.25^ab^ (86.73)	64.03^b^ (88.67)
Soil fertility	Soil described as fertile (0/1)	0.41	0.31^a^	0.46^ab^	0.50^b^
***Community-level variables***					
Distance to Puyo	Time to reach Puyo from community center (minutes)	154 (202)	45^a^ (66)	135^b^ (84)	283^c^ (218)
Population density	Inhabitants/ km^2^ of community land	17.57 (28.65)	60.68^b^ (42.68)	11.58^a^ (14.03)	6.35^a^ (8.45)

Note: (0/1) identifies dummy variables. Standard deviations in parentheses for continuous variables. Values followed by the same letter are not significantly different from each other (p ≤ 0.05). While we take logs of the distance from dwelling to road, the total land available for use and the travel time to Puyo in the regression analysis, we provide non-logged values here for ease of understanding.

A methodological issue should be addressed before proceeding. Household decisions regarding land use may be influenced by contextual (community) variables, which are distinctive for each community, because households within a community may exhibit similar land use patterns based on a common background in terms of ethnicity, economic well-being, market integration and environmental endowments [12, 46]. Failing to control for the hierarchical nature of the data may lead to misleading interpretations of individual and household variables. In order to control for contextual effects on household land use decisions, we use a hierarchical model. In the case of total cultivated area or areas in staple or primarily subsistence crops, we use a multilevel linear model of the following form:

$$y_{ij}\;=\;\alpha +X_{ij}\beta +\varepsilon_{ij}+v_{j}$$ (1)

where *y*_*ij*_ stands for land use (either the natural logarithm of the total cultivated or the area in staples) of household *i* in community *j*, ***X*** is a vector of household and community-level covariates (described below), *β* is a vector of household and community coefficients, *ε*_*ij*_ is the household-level error term, and *v*_*j*_ is the community-level error term.

For the fractions of land in cash crops and in pasture, another issue must be addressed. Each of these dependent variables is continuous, taking on the value of zero for a large part of the sample (72% and 62%, respectively). Using a linear regression approach under such circumstances would result in inconsistent estimators. Instead, this kind of *corner solution outcomes* [51] can be modelled using a Tobit approach. So, to control for the censored nature of the dependent variables, we rely on a Tobit model of the following form:

$$y_{ij}*\;=\;X_{ij}\beta$$ (2)

$$y_{ij}\;=\;0\;\text{if}\;y_{ij}\leq 0$$

$$y_{ij}\;=\;y_{ij}*\;\text{if}\;y_{ij}*>0$$

where *y*_*ij*_ is the fraction of the total area devoted to cash crops or the fraction of the total area in pasture of household *i* in community *j*, *β* is a vector of household-level coefficients, ***X*** a vector of household and community-level predictors The observable dependent variable *y* equals 0 if the latent variable y*≤0 and will take the value of y* if y*>0. The results of a Tobit model cannot be directly interpreted since the coefficients show to what extent a change of one unit in ***X*** has on the latent variable y*. As we are interested in the effects of ***X*** on *y* rather than y*, we calculate the marginal effects for the unconditional value of *y*.

### Specification

Definitions and descriptive statistics are presented in Table 2. Household demographic characteristics include the age and education of the head as well as two dichotomous variables taking the value of 1 if the head is Kichwa or Shuar, respectively, with colonist households as the reference group. The model specification also includes household size since agricultural land use may be influenced by household labor availability.

Financial capital variables include household spouse. A wealth index was derived as the first principal component of the possession of a radio, television, cell phone, computer, gas stove, refrigerator, chainsaw, spray pump, car, motorcycle, solar panel, boat and rifle This accounted for 29% of the variation in the data. The methodology above referred to is preferred since, in contrast to simple count indices, which assign equal values to every asset, it gives higher weight to the assets which provide more information about household wealth [52]. There is also the possibility that reverse causality exists between wealth and land use decisions, resulting in endogeneity. However, this possibility is low here, as the wealth index is constructed upon assets accumulated over a relatively long period of time which greatly reduces the likelihood of reverse causality. Another dummy variable indicates whether the household has any member engaged in off-farm employment, to control, for this potentially important effect of income diversification.

We include the natural logarithm of distance to the closest road in the list of predictors since this variable reflects the ease of marketing agricultural products and hence should be an important determinant of land use decisions, as found in previous work [53, 54]. The number of times that a household participated in community work activities (*mingas*) during the preceding 12 months provides an indicator of social capital, which may affect the dependent variables. The time the household head has resided in the community may influence the cultivated area since it reflects the “household life cycle”, with longer-term residents having more time to clear larger areas [55]. Thus, in the commonly observed “peasant pioneer cycle”, the new settler first focuses on subsistence crops (to survive), then clears more land to add cash crops, and then later clears more to diversify into pasture for cattle [5]. To control for this potential source of variance, two dummy variables are created, the first taking the value of 1 if the duration time of residence is short (under 10 years), and the second one taking the value of if it is medium-term (see Table 2 for definitions), with long-duration of 25 + years as the reference category.

The household’s natural capital endowment is taken to be the natural logarithm of the amount of land owned or controlled via common property allocation. Additionally, we add a dummy variable to the model to indicate if the plot is reported by the respondent to have fertile soil. Finally, while the type of land tenure may be an important determinant of land use [47, 56], we do not control for this variable as it is closely linked to ethnicity, with all colonist households having private rights and all indigenous households holding common property rights over lands allocated to them by the community *asamblea* (assembly of all household representatives). In this sense, it is worth noting that all indigenous communities in our sample had recognized collective usufruct rights over their lands, while all colonist households declared having legal rights over the land they possessed. This means they can freely use those lands for agriculture and hunting, but cannot sell any part of the land or extract trees without permission of the *asamblea*, though unauthorized felling of trees is not uncommon.

At the community-level, the natural logarithm of the time required to reach Puyo (the provincial capital and by far largest town in the area) is used to control for the level of market access, which is likely linked to larger areas in crops (the closer the household’s farm to the road and to Puyo, the larger the area in crops). Additionally, as the availability of natural resources including land may be limited by population pressure [28, 43], population density at the community-level (inhabitants per square kilometer) is included in the model specification. Preliminary specifications considered several other household-level predictors, including dummy variables for female headed households; on whether the household received the governmental transfer payment for poor households with small children (*Bono de Desarrollo Humano*); and on whether the household had received credit from a bank or other source. Finally, distance from the dwelling to the household’s largest agricultural plot was examined on the hypothesis that a longer distance would lead to less intense use of that plot. These predictors were dropped from the final model specification, however, as they did not contribute to the model fit.

## Results and discussion

### Descriptive analysis

In Table 2, we compare the means of the independent and dependent variables using one-way ANOVA and pairwise comparison of means. Values followed by the same letter are not significantly different from each other (p ≤ 0.05). The means of the fraction of land devoted to agriculture (crops and pasture) for colonists and the Shuar are not significantly different from each other. In contrast, the Kichwa devote a smaller part of their land to agricultural purposes. In terms of staple and cash crops, there are not significant differences across ethnic groups. The mean fraction of the land devoted to pasture of Kichwa households is significantly smaller than that of colonist and Shuar households. It is worth noting, however, that this descriptive analysis does not include multivariate controls. In the following section, we incorporate sets of household and community predictors to disentangle the effect of ethnicity on agricultural land use.

Table 2 also shows the mean values of the independent variables for the three ethnic groups. Shuar heads are younger and, interestingly, have, on average, more years of formal education than their colonist and Kichwa counterparts. Kichwa households are significantly larger than their Shuar and colonist peers. A possible explanation is that migration to urban areas, other provinces or even abroad is reported to be a common strategy of both colonists and Shuar to cope with land scarcity and crop failures [26]. However, a more likely one is differences in fertility, based on data from the northeast Ecuadorian Amazon where the Shuar and Kichwa both have much higher fertility than colonists, but the Shuar have more out-migration than the Kichwa [30, 57]. Whereas it is difficult to interpret the values of the wealth index, the negative sign for the Kichwa may reflect that they are poorer in terms of the assets upon which the index was constructed [58]. In terms of off-farm employment, Kichwa households are the least likely to engage in it among the three ethnic groups, which may be related to Kichwa households choosing to live farther away from urban areas [29].

With respect to the remaining household-level variables in the model. The Shuar and the colonists live much nearer to roads than the Kichwa. This is probably related to colonists living closer urban areas, where road density is higher, and the Shuar tendency to settle near roads rather than fruit orchards [57]. On average, Shuar households also take part in community meetings/work significantly more often than Kichwa and colonist households, reflecting perhaps closer community ties, at least in this area. The durations of residence time variables show interesting patterns. The shares of households with short, medium and long durations of residence in their present community are quite similar for colonist and Shuar household heads, while in contrast the vast majority of Kichwa household heads (four out of five) have lived in the community for a long time (over 25 years). As noted above, the Shuar are originally from the southern Ecuadorian Amazon and started migrating north pushed by high rates of population growth leading to increasing land scarcity and by crop failures. In fact, 61% of the Shuar heads were born in the province of Morona Santiago. Land sizes differ significantly across the three populations, with Kichwa households having over double the land of Colonists, and the Shuar right in the middle (mean values being 64 ha, 28 ha, and 45 ha, respectively). In terms of soil quality, soils are reported by colonist respondents to be less fertile than those of Kichwa and Shuar respondents, though this is highly subjective and could be colored by colonists coming from areas of the country with better soils for agriculture (e.g., the Sierra highlands, the origin of about 80% of colonists).

Finally comparing the values at the community-level, colonist households live much closer to Puyo, the provincial capital, with Kichwa households farthest away (more than six times as far), and Shuar villages in between. It is also worth noting that population density is much larger in colonist areas than in the sample Kichwa and Shuar communities.

### Multivariate findings

This section presents the main empirical findings of this research, based on pooling data for all interviewed households of the three ethnicities and controlling for ethnicity by the use of dummy variables. The results are presented in Table 3 for the four dependent variables which reflect different aspects of land use. Before proceeding we must clarify that the results presented here are based upon cross-sectional data, so that they cannot be interpreted as causal effects. First, there is a nonlinear relationship between age and the fraction of land devoted to agriculture, which is captured by the (positive) age and (negative) age squared variables. Thus, the share of land used for agricultural purposes rises with age up to some age, then declines, with the turning point occurring at 56 years. This is consistent with prior research [12] which found that total cultivated area is larger for households with older heads.

**Table 3:** Determinants of total agricultural area, and shares of area in staple crops, cash crops and pasture.

	Total agricultural area	Staple crops	Cash crops	Pasture
	Multilevel linear model		Multilevel Tobit (marginal effects)	
Age	0.013***	0.003	0.001	0.003
Age squared	-0.000***	-0.000	-0.000	-0.000
Education	-0.001	0.000	0.000	0.003
Kichwa (0/1)	-0.000	0.037	-0.019***	-0.111***
Shuar (0/1)	0.039	0.035	-0.010*	0.012
Household size	0.007	-0.004	0.001	-0.002
Wealth index	0.014*	-0.001**	0.000	-0.012*
Off-farm employment (0/1)	-0.020	0.011	-0.010**	-0.069**
Distance from dwelling to road	-0.026**	0.015	-0.008***	-0.041***
Participation in social events	0.000	0.000	0.000	-0.000
Short duration of residence (0/1)	-0.009	0.072***	0.000	0.022
Medium duration of residence (0/1)	-0.021	-0.035	-0.001	0.008
Soil fertility (0/1)	0.000	0.000	0.009	0.001
Total land area available for use	-0.193***	-0.105***	-0.001	0.036***
Distance to Puyo	0.030	0.00	0.005	-0.003
Population density	-0.001***	0.000	-0.000*	0.000
Intra-class correlation	0.173	0.140	-	-
Wald test	517***	262***	48***	108***
Number of observations	304	304	304	304

Note:

*, ** and *** indicate statistical significance at 10% (marginal), 5% and 1% levels, respectively.

In terms of the key effects focused on in this paper, ethnicity is observed to have no effect on either the overall fraction of land devoted to agriculture and the fraction of land dedicated to staple crops. In the first case, this finding is not consistent with the descriptive evidence presented in Table 2 but here we are controlling for other factors. In contrast, having a Kichwa head significantly reduces the share of land devoted to both cash crops and pasture. Having a Kichwa head reduces the fraction of land in crops and pasture by 2 and 11% respectively.

Continuing with other variables, at the household level, wealth (assets) is negatively correlated with the fraction of land dedicated to staple crops. A probable explanation is that wealthier households can purchase staple foods (plantains and cassava) as well as a broad variety of other foods in the market whereas poorer households need to rely more on what they can produce themselves. Off-farm employment also plays a significant role in agricultural land use: Receiving off-farm income reduces the fraction of land devoted to cash crops and pasture by 1% and 7%, correspondingly. There are two likely explanations: First, off-farm earnings in the Ecuadorian Amazon tend to be higher than those from agriculture [29], so that off-farm earnings relax household needs to clear forest and expand the agricultural area or to work intensively on the farm. A second possibility has to do with the opportunity cost of time, since having off-farm employment especially in the form of a permanent job (e.g., public employee, school teacher, waitress, soldier, among others) greatly reduces the time an off-farm worker has to work on the farm [54, 59, 60].

The distance to the closest road is linked to a lower fraction of land devoted to agriculture, specifically to less land in cash crops and pasture. Establishing cash crop plots near roads facilitates the transport of inputs to the field and taking the produce to the road to transport to markets [61]. This finding is consistent with previous research [53, 54, 62, 63] showing that forest clearing occurs mostly near roads, where agriculture flourishes. This is also consistent with the theory of von Thunen [64]. Note that there is no effect of road access on the area in staple crops, precisely because most is consumed by the household. Also, once other factors are controlled for, social capital does not play any role in shaping land use decisions, though no doubt the effects are positive on the quality of life (mingas are common among both Kichwa and Shuar but not colonists) [65, 66]. Among the duration of residence variables, compared to the long duration of residence, the results for the short duration dummy variable show that in the early years the household focuses on staple crops, which is consistent with the “farm lifecycle” theory (as well as the “peasant pioneer cycle”) documented in the northern Ecuadorian Amazon by [5, 31], with initial settlement involving the establishment of crops to cover immediate food needs.

The final two household-level variables refer to the role of natural resource endowments or natural capital—the area of land perceived as one’s own (even if communal land allocated by the community *asamblea* in the cases of the Shuar and Kichwa) and its perceived soil quality. One expects natural capital to be positively linked to the area in use, since a larger area or quantity of land available facilitates a larger area in use, and land of better quality produces more for a given labor effort. Indeed, in the study population, we see from Table 3 that a larger *quantity* of land owned or available to a household is indeed linked to a smaller *share* of land used for agriculture and for staple crops. This reflects that households with less land tend to use it more intensively [5]. At the same time, larger farms are associated with larger shares of land in pasture. These findings are consistent with those [5] for colonist households in the Northern Ecuadorian Amazon, and may be related to the fact that pasture yields in the Amazon are very low so that large areas are needed for grazing cattle (about 1 ha per cow) [67].

On the other hand, we did not find any significant correlation of perceived land *quality* on the outcome variables. On the one hand, one might think this is due to the subjective (and not necessarily correct) nature of the response, or perhaps the wide variability of soil qualities observed on Amazonian farms in Ecuador. But in the case of migrant colonists in the northern Ecuadorian Amazon, a positive statistically significant relationship was found between (perceptions of) soil quality and area of land cleared and in agricultural use [5, 46]. Thus, the lack of any significant relationship here for these populations suggests that indigenous farmers do not pay much attention to (or observe accurately) soil quality, since many of their main crops (cassava, cacao) grow adequately in mediocre soils anyway.

In concluding with respect to the community level factors, once other factors have been controlled for, it was surprising that distance along the road to the main city and market (Puyo) is not negatively related to the area in agricultural use as expected. One possible explanation is that other predictors (i.e., ethnicity, distance from household to road and population density) already capture this effect. However, the effect of distance to Puyo remains non-significant even when these three predictors are removed, which leads us to think that distance to markets does not play a role in shaping land use decisions. Finally, population density in the community/vicinity has a statistically significant *negative* relationship to the fraction of land devoted to agriculture, which may reflect farm sizes shrinking over time near Puyo as a result of high rates of population growth and resulting subdivision of plots among heirs, as has occurred among both colonists and indigenous populations elsewhere in the Ecuadorian Amazon [12, 30]. It also might reflect increasing economic diversity and therefore greater access to off-farm work in larger, denser communities (and better bus linkages to Puyo). In any case, this linkage does not reflect causality but rather the joint consequences of growing populations.

## Concluding remarks

This paper is unusual in documenting differences in land use and investigating the factors responsible for these differences among both migrant settlers and indigenous populations based on collecting identical data on both types of populations in a single survey. The results are generally consistent with prior research showing that some indigenous peoples (e.g., Shuar) are becoming increasingly integrated in the market economy, engaging in cattle ranching and commercial agriculture, and becoming more like migrant colonists [10–12, 14]. However, there is much variation in land use among and within indigenous groups [27], with many Kichwa communities in this study retaining their traditional agricultural practices based on subsistence agriculture.

Our findings thus empirically confirm that there are important differences in land use among indigenous groups in the Amazon, with the Shuar sharing several key characteristics of colonists, first, in establishing communities near roads, facilitating diversifying income sources, and engaging in commercial cash crop production and cattle ranching. Furthermore, most Shuar are also migrants, moving to Pastaza, pushed by population growth, land scarcity and livelihood risks in their original territories in the southern Amazon. it is therefore not surprising that the Shuar and colonists exhibit somewhat similar land use patterns. However, prior research [10] depicted a process of convergence in agricultural/land use patterns between the Shuar and mestizo colonists, which was followed by a process of divergence, with the Shuar reforesting and using more sustainable agricultural practices (i.e., home gardens with cocoa and coffee as cash crops). It is also worth noting that unsustainable land use practices have also been reported for other indigenous groups, such as the Secoya of the northern Ecuadorian Amazon during the 1990s [12]. Also in the northern Ecuadorian Amazon, Vasco et al. [68] found that Kichwa households are as likely as mestizo colonists to engage in illegal logging. These examples as well as this present work suggest that different ethnic groups exhibit different land use patterns depending on local conditions, migratory and settlement histories and location, level of exposure to markets, and external factors. Thus, indigenous stewardship of natural resources cannot be taken for granted, and policy interventions, including perhaps payments for ecosystem services programs, creation of alternative income sources (such as ecotourism, public or private sector non-agricultural employment), and promotion of more land-intensive agriculture) should also be considered in indigenous territories.

In this context, it is also worth revisiting the role of accessibility, as it appears more complex than has been understood before. First, it has several dimensions, including distance of the farm household to the nearest road and distance from that point to the major urban cynosure or market in the region. While no significant effect of the latter is found here, the areas cultivated in cash crops (including *naranjilla*) and in pasture are larger in the proximity of roads. This suggests that, in this Ecuadorian Amazon context, the distance to a road matters more than the distance to markets in shaping land use decisions.

In the last two decades, the Government has carried out significant investments in road infrastructure in the Amazon [69], which have facilitated population mobility and increased off-farm work opportunities in urban areas (including public sector employment). However, the expansion of the road network and paving of roads has also triggered further conversion of forest to agricultural land. While some advocate for restricting the construction of new roads in the Amazon in general [5], governments (national, provincial, municipal) generally see the expansion and improvement of the road network as a development priority [36, 70]. Indigenous peoples themselves have mixed perceptions, with some preferring staying isolated from markets and rejecting the construction of roads in their territories, and others, principally those living in communities close to already existing roads, actively seeking more roads [71, 72]. This poses a major challenge for practitioners seeking to balance rural development and conservation policies in indigenous territories.

Off-farm employment may help achieve both more rural development and poverty reduction and conservation in the Amazon since it provides higher incomes while reducing household labor allocated to farm work. However, it is worth noting that well-paid and stable off-farm jobs are found principally in the public sector and greatly depend on the country’s economic performance [29]. Given the country’s economic downturn in the last years, one can still wonder how sustainable it is in the long run. With this background, policies should be oriented to promote (only) environmentally friendly entrepreneurial activities such as ecotourism, community-based tourism, scientific tourism and processing and industrialization of renewable forest products. Since these entrepreneurial ventures are expected to benefit large share of the population, policies should especially encourage and support community-based initiatives. In this sense business training and credit for community-based enterprises may be very helpful in achieving that goal.

## Supporting information

S1 File Data base. Data base utilized for the multivariate analyses.
(DTA)

S2 File Ethics committee approval. Confirmation that the survey model used for this manuscript was approved by the Ethics Committee at Universidad Estatal Amazónica.
(PDF)
