# Supplementary material for: Agricultural land use among mestizo colonist and indigenous populations: Contrasting patterns in the Amazon
Source: PLoS One. 2018 Jul 5;13(7):e0199518. doi: 10.1371/journal.pone.0199518 (PMC6033409; doi:10.1371/journal.pone.0199518)
Supplement: S2 File — Confirmation that the survey model used for this manuscript was approved by the Ethics Committee at Universidad Estatal Amazónica. (PDF) [file pone.0199518.s002.pdf]

TO WHOM IT MAY CONCERN

This is to confirm that the survey template utilized in the framework if the research project entitled "livelihoods Strategies in the Pastaza province" was approved by the ethics committee at the Universidad Estatal Amazónica at Puyo, Ecuador on 17 December 2012.

The protocol utilized to conduct surveys in rural communities in Pastaza is as follows:

In order to collect data in a community, the survey leader approached the community leader in order to inform him/her about the nature and characteristics of the study, and to get his/her permission to conduct the survey. No survey was done without asking permission from the community leader.

Besides that, the first question of the survey model had a question asking for interviewees' oral approval to participate in the study. All interviewees were asked for oral approval before conducting the survey.

Sincerely,

Dr. C. Julio César Vargas Burgos, PhD.
RECTOR DE LA UNIVERSIDAD ESTATAL AMAZÓNICA
JVB/MBS
